# Mechanisms, strategies, and clinical application progress of subcutaneous transplantation angiogenesis

**DOI:** 10.3389/fbioe.2025.1665643

**Published:** 2025-11-19

**Authors:** Yiwen Li, Xuejin Ye, Sheng Chen, Lin Guo, Zhiran Xu, Jibing Chen, Hongjun Gao

**Affiliations:** 1 Ruikang Hospital Affiliated to Guangxi University of Chinese Medicine, Nanning, China; 2 GuangXi University of Chinese Medicine, Nanning, China

**Keywords:** angiogenesis, co-transplantation of cells, growth factors, immunomodulation, islet transplantation, scaffold materials, subcutaneous transplantation

## Abstract

Subcutaneous transplantation, as an important technology in cell and tissue engineering, has received considerable attention due to its simplicity of operation, strong reproducibility, and potential clinical application value. However, the limitations of the vascular network in subcutaneous tissue severely restrict the survival and functionality of transplanted cells; therefore, angiogenesis has become a key factor in improving the success rate of transplants. Currently, despite progress in the research of subcutaneous transplantation, there are still many challenges and shortcomings. This article reviews the molecular mechanisms of angiogenesis in subcutaneous transplantation, strategies involving cells and biomaterials, as well as the latest technological advancements in promoting angiogenesis. It focuses on analyzing research results in aspects such as growth factor delivery, co-transplantation of cells, scaffold material optimization, and immune regulation. At the same time, the article systematically summarizes the clinical application prospects and challenges of subcutaneous angiogenesis strategies in islet transplantation, soft tissue repair, and autoimmune diseases. By comprehensively analyzing the current research hotspots and difficulties, it aims to provide theoretical support and practical guidance for future basic research and clinical translation of angiogenesis in subcutaneous transplantation.

## Graphical Abstract

**Figure:**
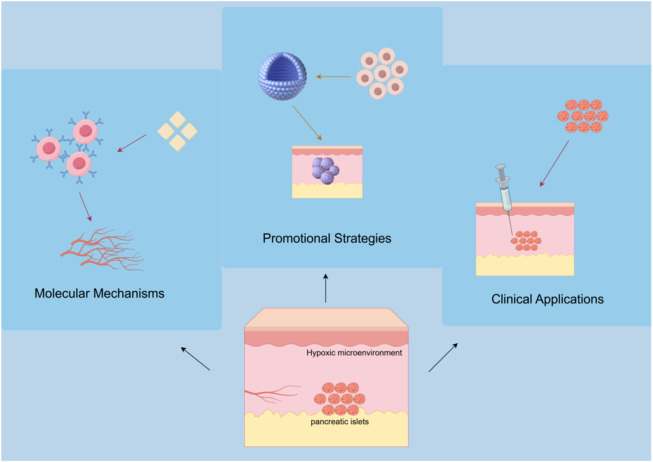

## 1 Introduction

In the field of subcutaneous transplantation, the mechanisms, strategies, and applications of angiogenesis have gradually become an important research direction. Subcutaneous transplantation is regarded as an ideal transplantation site for cell and tissue engineering due to its simplicity, ease of monitoring, and sampling. However, the subcutaneous tissue is characterized by sparse vascularity and a hypoxic microenvironment, as demonstrated by studies specifically measuring oxygen tension in this region (Mitsugashira et al., 2022). Histological and functional analyses have confirmed that the vascular density in subcutaneous areas is significantly limited (Jeon et al., 2024), and direct oxygen measurements consistently show lower oxygen partial pressures compared to other transplantation sites, which are critically associated with poor islet survival and function (Komatsu et al., 2016; Mitsugashira et al., 2022). Research indicates that angiogenesis is critical to the success of subcutaneous transplantation; the lack of effective vascular formation limits the survival and function of transplanted cells (Komatsu et al., 2021; Saito et al., 2024). In recent years, with the development of biomaterials science, stem cell technology, and molecular biology, strategies to promote subcutaneous angiogenesis have continuously enriched, providing new possibilities for clinical applications.

An important mechanism of angiogenesis during subcutaneous transplantation is to promote the formation of new blood vessels by regulating the microenvironment. Studies have shown that cytokines such as vascular endothelial growth factor (VEGF) play a key role in angiogenesis by stimulating the proliferation and migration of endothelial cells, thereby promoting the formation of new blood vessels (Kuwatsuka et al., 2024; Ma et al., 2025). Additionally, the type and source of stem cells significantly affect angiogenesis. For example, adipose-derived stem cells (ADSCs) can effectively promote angiogenesis and enhance transplantation survival rates after transplantation (Saito et al., 2024).

The application of biomaterials is particularly important in strategies to promote angiogenesis. Research has shown that specific biomaterials such as gelatin hydrogels (GHNF) can significantly enhance angiogenesis in subcutaneous transplantation by providing a supportive matrix and releasing growth factors to promote the formation of new blood vessels and the survival of cells (Saito et al., 2023b; Kranjc Brezar, 2024). For example, loading basic fibroblast growth factor (bFGF) into biomaterials can enhance the angiogenesis at the transplantation site, thereby improving the survival and function of transplanted cells (Duan et al., 2024; Ma et al., 2025). However, several investigations have indicated that the enhancement of angiogenesis by GHNF is somewhat constrained, and its fundamental mechanism does not primarily involve the direct stimulation of significant neovascularization (Kanai et al., 2023; Saito et al., 2023a). Instead, its principal function lies in safeguarding islet viability by bolstering the accumulation of extracellular matrix components (such as laminin, collagen III, and IV), rather than fostering angiogenesis.

Despite certain advancements in the strategies for promoting angiogenesis in subcutaneous transplantation, there are still challenges. For instance, the survival rate of transplanted cells *in vivo* is usually low, and the angiogenesis process after transplantation is influenced by various factors, including immune responses and changes in the microenvironment (Kuwatsuka et al., 2024; Zhou et al., 2024). Therefore, future research needs to further explore how to optimize these factors to improve the success rate of subcutaneous transplantation.

In summary, the mechanisms and strategies of angiogenesis in subcutaneous transplantation represent a complex and significant research area. By deeply understanding the molecular mechanisms of angiogenesis and applying advanced biomaterials, it is hoped that more effective clinical treatments for subcutaneous cell and tissue transplantation can be achieved in the future.

## 2 Main body

### 2.1 Molecular mechanisms of angiogenesis in subcutaneous transplantation

#### 2.1.1 Basic biological processes of angiogenesis

Angiogenesis is a complex biological process involving interactions between various cell types, primarily including the activation, proliferation, migration of endothelial cells, and the formation of lumens. The main driving factors of angiogenesis are the actions of various growth factors, particularly vascular endothelial growth factor (VEGF) and basic fibroblast growth factor (bFGF), whose release can promote the proliferation and migration of endothelial cells, further facilitating the formation of new blood vessels. VEGF initiates intracellular signaling pathways by binding to its receptors, activating a series of downstream effects, thus stimulating the proliferation and migration of endothelial cells (Ferrara, 2004).

**FIGURE 1:**
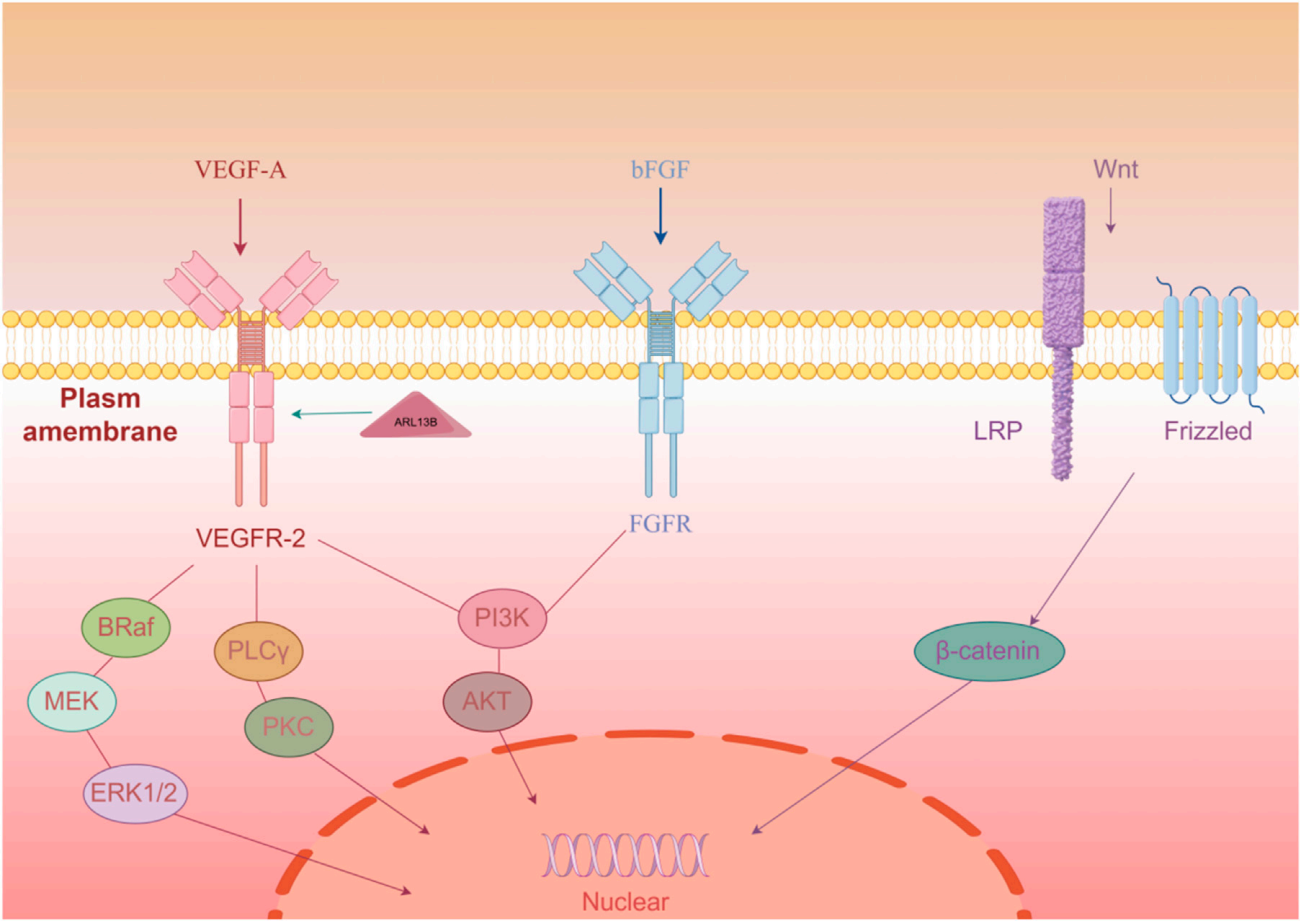
Three key factors (VEGF-A, bFGF, Wnt) initiate signaling through membrane receptors, activating downstream pathways to regulate angiogenesis-related gene expression.

In clinical and experimental research on subcutaneous transplantation, the regulation of the WNT/β-catenin signaling pathway has been found to play an important role in promoting angiogenesis. Studies have shown that activating the WNT signaling can enhance angiogenesis at the subcutaneous transplantation site, thereby improving the survival and function of the transplanted tissue (Wang et al., 2024). Therefore, by using WNT agonists, it is possible to effectively promote the level of angiogenesis at the transplantation site, providing new therapeutic strategies for the clinical application of subcutaneous transplantation (Wang et al., 2024). This regulatory mechanism involves the proliferation and migration of vascular endothelial cells and is closely related to cell signaling interactions in the local microenvironment, highlighting the potential application value of the WNT/β-catenin signaling pathway in subcutaneous transplantation (Figure 1).

In the process of angiogenesis, microenvironment factors, such as hypoxic conditions, often upregulate the expression of these factors, creating a favorable growth environment that further promotes angiogenesis. For example, hypoxia-inducible factor (HIF) can be activated under hypoxic conditions, leading to an increase in the expression of pro-angiogenic genes, thereby promoting the formation of new blood vessels (Ruas et al., 2007). Additionally, mesenchymal stem cells (MSCs) are believed to play a central role in this process. Studies have shown that MSCs promote local angiogenesis and tissue healing by secreting various growth factors and regulating the behavior of surrounding cells (Ma et al., 2022).

#### 2.1.2 Role of VEGFA-VEGFR2 signaling pathway in subcutaneous angiogenesis

VEGFR2, as an important signaling transduction receptor, plays a crucial role in angiogenesis. VEGFA binds to VEGFR2, activating multiple downstream signaling pathways, including PI3K/Akt and MAPK/ERK pathways, which can promote the proliferation and migration of endothelial cells, thus facilitating the formation of new blood vessels (Xu et al., 2017; Tanaka et al., 2020). At the same time, the expression of ARL13B protein is closely related to the localization of VEGFR2; research has found that modulation of ARL13B activity can enhance VEGFR2 function, further promoting the proliferation and migration of endothelial cells, thereby increasing the efficiency of angiogenesis (Chen et al., 2023).

#### 2.1.3 Hypoxia-inducible factor (HIF) and local microenvironment regulation

The hypoxic conditions in the local microenvironment play a critical role in restoring and promoting angiogenesis. Studies have shown that hypoxic states can activate the HIF signaling pathway, thereby promoting the expression of genes related to angiogenesis, including the upregulation of growth factors like VEGF and bFGF. This process can promote the release of endogenous angiogenic factors, further improving the hemodynamic status of the tissue and promoting tissue healing and regeneration (Zou et al., 2012; Zhang et al., 2020). Research has also indicated that MSCs promote angiogenesis through interactions with the local microenvironment and regulate immune responses, creating a favorable environment for wound healing (Kananivand et al., 2025; Zhang et al., 2025).

### 2.2 Growth factor delivery strategies to promote angiogenesis

#### 2.2.1 Local delivery of bFGF and its effects

Basic fibroblast growth factor (bFGF) is widely regarded as a key factor in promoting angiogenesis, playing an important role in the proliferation of endothelial cells and the formation of vascular networks. The pro-angiogenic potential of bFGF was first established in early foundational studies, which directly demonstrated its ability to stimulate the proliferation and migration of endothelial cells, laying the groundwork for its role in vascular formation (Ratajska et al., 1995). In the context of controlled-release delivery, early technologies emerged in the form of heparin-gelatin microspheres and PLGA scaffolds, which successfully achieved sustained release of bFGF and were validated to induce new blood vessel formation in subcutaneous models (Tabata et al., 2000). Furthermore, pre-vascularization concepts were pioneered through these systems, where bFGF-controlled release was used to establish functional vascular networks in subcutaneous tissues, providing critical support for subsequent tissue engineering applications (Tabata et al., 2000; Gardner-Thorpe et al., 2003).

Subsequent research further confirmed that bFGF promotes the formation of new blood vessels by stimulating the proliferation and migration of endothelial cells, thereby aiding the healing of injured tissues. For example, in a study on bFGF, it was shown that bFGF can effectively promote the proliferation and tubular structure formation of human umbilical vein endothelial cells (HUVECs), which are crucial for angiogenesis (Lei et al., 2024). Additionally, bFGF enhances the survival of endothelial cells by activating multiple signaling pathways (such as PI3K/Akt and MAPK/ERK), thereby further promoting angiogenesis (van Velthoven et al., 2023).

To improve the biological stability of bFGF and reduce its rapid degradation in the body, researchers have explored strategies to combine bFGF with biodegradable materials, such as collagen gelatin sheets. Current research on bFGF focuses on several key areas: carrier technology innovation, with recent studies developing novel delivery systems (e.g., electrospun fiber membranes) that enhance pro-angiogenic effects by sustained release of Cu^2+^ or regulating autophagy (Yang A. et al., 2025); microenvironment regulatory mechanisms, including exploring new functions in tumor microenvironments (e.g., CAFs secreting IL-6) and immune regulation (e.g., macrophage polarization) (Yi et al., 2024; Zhang et al., 2024; Ly et al., 2025); and expanded disease applications, covering complex pathological models such as diabetic wound healing and pancreatic cancer immunotherapy. By loading bFGF into collagen gelatin sheets, sustained release can be achieved, providing long-term bioactivity in the transplantation area. This approach effectively prolongs the action time of bFGF and enhances its angiogenesis effect in the transplantation area. In a study, subcutaneous transplantation using bFGF-loaded collagen gelatin sheets resulted in a significant increase in angiogenesis in the transplantation area, promoting blood supply and healing of surrounding tissues (Shen et al., 2022).

Moreover, the sustained release of bFGF also helps regulate the local microenvironment, promoting the expression of endogenous angiogenic factors, thereby further enhancing the angiogenesis effect. Through such a biomaterial delivery system, the clinical application potential of bFGF has been greatly enhanced, providing new solutions for recovery after transplantation surgery. The effectiveness of this strategy has been validated in several animal experiments, indicating that the combination of bFGF and biodegradable materials can play an important role in tissue regeneration and repair (Syromiatnikova et al., 2024; Sun et al., 2025).

#### 2.2.2 Nanogel technology

Nanogels, as emerging drug delivery carriers, have gained widespread attention due to their unique physicochemical properties and biocompatibility. In recent years, researchers have explored various preparation methods for nanogels, particularly the application of nanogels using semipermeable membrane technology in drug delivery and tissue engineering. These nanogels typically possess good biocompatibility and adjustable drug release characteristics, making them suitable for use in fields such as gene delivery and regenerative medicine.

Nanogel synthesis can be achieved through various methods, including self-assembly, polymerization reactions, and cross-linking. Among them, polymer-based nanogels are favored due to their outstanding biocompatibility and biodegradability. For example, the application of nanogels has demonstrated excellent drug loading capacity and release performance, effectively responding to tumor microenvironments and releasing drugs (Gülyüz et al., 2024). Additionally, nanogels made from temperature-responsive materials (such as poly (N-isopropylacrylamide), PNIPAM) can regulate their swelling degree under specific temperature conditions, thus achieving controlled drug release (Niezabitowska et al., 2023; Vashist et al., 2024). Future research could explore the synergistic effects of such nanoparticle gel localized controlled release systems with angiogenic factors (such as VEGF).

In summary, nanogels provide new ideas for the design of drug delivery systems. As the understanding of these materials deepens, future research will focus on enhancing the targeting ability and biocompatibility of nanogels to promote their translation in clinical applications. At the same time, the multifunctionality and intelligent response characteristics of nanogels are sure to open new pathways for personalized medicine and precision therapy.

#### 2.2.3 Platelet-rich plasma (PRP) assisted promotion of angiogenesis

Platelet-rich plasma (PRP) is an autologous blood product that has attracted attention in regenerative medicine due to its rich content of growth factors. In recent years, increasing research has shown that PRP can effectively promote angiogenesis and demonstrate good effects in various clinical applications. One of the main mechanisms of action of PRP is the release of growth factors, such as platelet-derived growth factor (PDGF), transforming growth factor β (TGF-β), and VEGF, which play important roles in promoting cell proliferation, migration, and angiogenesis (Guo et al., 2017; Zhou et al., 2017). Particularly in fat grafting surgeries, the application of PRP can significantly improve the survival rate of adipose tissue and enhance blood supply to the graft site, thereby promoting healing and regeneration (Oh et al., 2011).

In clinical research, the efficacy of combining PRP with fat grafting has been validated. For instance, studies have shown that the combination of PRP and fat grafting can significantly enhance the survival rate of transplanted fat and promote the formation of new blood vessels, an effect primarily attributed to the abundant growth factors in PRP (Zhou et al., 2017). Additionally, the activating effect of PRP on endothelial cells has also been widely studied, with results indicating that PRP can promote the proliferation and migration of endothelial progenitor cells, thereby accelerating the angiogenesis process (Zhang et al., 2019).

Further research has also found that the angiogenic effects of PRP vary depending on the preparation methods. For example, PRP activated by specific methods has shown better results in promoting the release of growth factors and enhancing cell viability (Kakudo et al., 2014; Zhou et al., 2017). Furthermore, the composition and concentration of PRP can also affect its angiogenic effects. Studies have indicated that appropriate platelet concentrations and suitable activation methods can optimize the biological activity of PRP, thereby more effectively stimulating angiogenesis and tissue repair (Guo et al., 2017).

At the same time, PRP has shown good application prospects in treating chronic wounds and diabetic foot ulcers. Research has found that PRP can promote angiogenesis by regulating the secretion of cytokines, thus accelerating wound healing (Zhang et al., 2019). In these cases, PRP not only enhances angiogenesis but also promotes tissue regeneration by improving the local microenvironment, demonstrating its broad application potential in regenerative medicine (Xu et al., 2020).

In summary, platelet-rich plasma (PRP), as a biologically active agent rich in growth factors, has shown exceptional application value in multiple clinical areas by enhancing angiogenesis and improving fat survival rates. Future research should continue to explore the optimal preparation and application methods for PRP to maximize its potential in regenerative medicine.

### 2.3 Stem cells and co-transplantation strategies

#### 2.3.1 The role of mesenchymal stem cells (MSCs) in promoting angiogenesis

Mesenchymal stem cells (MSCs) play an important role in promoting angiogenesis. Research shows that MSCs can secrete various angiogenic factors, such as VEGF, matrix metalloproteinases (MMPs), and epidermal growth factor (EGF), which promote angiogenesis by regulating endothelial cell proliferation, migration, and lumen formation (Ma et al., 2022). Additionally, MSCs can enhance the effects of angiogenesis by altering the local immune environment. For instance, MSCs can promote the polarization of M2 macrophages, which have been shown to play a key role in wound healing and angiogenesis (Mohamad Yusoff and Higashi, 2023). This polarization mechanism not only helps to reduce local inflammatory responses but also promotes the formation of blood vessels by secreting more cytokines and growth factors.

In clinical applications, the co-transplantation of MSCs with other cell types (such as β cells) is considered an effective strategy to enhance cell survival and functional recovery. Studies show that the combined transplantation of MSCs and β cells can significantly improve the survival and function of transplanted cells, especially in diabetic models, where MSCs provide favorable conditions for β cell survival by improving the microenvironment and promoting angiogenesis (Yu et al., 2020). Furthermore, MSCs also show promising prospects in wound healing caused by diabetes, as they accelerate the wound healing process by promoting local blood flow and nutrient supply (Huang et al., 2022).

Moreover, as research delves deeper into the biological characteristics of MSCs, increasing evidence supports their potential in treating various vascular-related diseases. For example, studies have found that MSC-derived exosomes not only promote angiogenesis but also regulate inflammatory responses, which may provide new insights for treating chronic wounds and cardiovascular diseases (Liu and Liu, 2022). These findings indicate that the role of MSCs in promoting angiogenesis extends beyond direct cellular activities, encompassing intercellular signaling and microenvironment regulation to achieve more complex biological effects.

Overall, the role of MSCs in promoting angiogenesis is multifaceted, involving the secretion of cytokines, regulation of the immune environment, and interactions with other cell types. These characteristics make MSCs an important research focus in the field of regenerative medicine and provide new treatment strategies for ischemic diseases, diabetic complications, and more. As research progresses, the clinical application prospects for MSCs will further expand, potentially playing a greater role in the treatment of angiogenesis-related diseases.

#### 2.3.2 3D cell spheroid technology

In the fields of tissue engineering and regenerative medicine, the application of three-dimensional (3D) cell spheroid technology has gained increasing attention. Compared to traditional two-dimensional (2D) cell culture, 3D cell spheroids can better mimic the microenvironment of cells *in vivo*, allowing cells to grow and interact under conditions that are closer to physiological states. This technology is particularly suitable for the co-culture of human umbilical cord blood mesenchymal stem cells (MSCs) and human umbilical vein endothelial cells (Steiner et al., 2022), constructing a vascularized three-dimensional microenvironment that helps promote the angiogenesis and survival of transplanted β cells.

In studies, 3D cell spheroids constructed using human umbilical cord blood MSCs and human umbilical vein endothelial cells have demonstrated significant angiogenic capabilities (Steiner et al., 2022). Specifically, these cell spheroids can effectively promote the proliferation and migration of endothelial cells, enhancing blood vessel formation. This process is crucial for the survival of transplanted β cells, as they require adequate blood supply to maintain their function and survival rates. Research indicates that the constructed 3D cell spheroids not only enhance cell survival but also effectively alleviate hypoxia during the transplantation process, further improving the success rate and functional performance of the transplantation (Duman et al., 2022).

In addition, a notable advantage of 3D cell spheroid technology is its ability to simulate intercellular interactions and signaling, which is difficult to achieve in 2D cultures. Direct contact between cells and the presence of extracellular matrix can more realistically reflect cellular behavior under physiological and pathological conditions. For example, within 3D spheroids, cells are better able to express genes related to angiogenesis, promoting the formation of new blood vessels (Mathot et al., 2021). This finding provides new insights for β cell transplantation, especially in the treatment of metabolic diseases like diabetes, showing promising application prospects by utilizing 3D cell spheroid technology to enhance the survival and function of transplanted cells.

Overall, the combination of human umbilical cord blood MSCs and human umbilical vein endothelial cells to construct 3D cell spheroid technology is not only significant in basic research but also offers new opportunities for clinical applications. With further research into this technology, future breakthroughs and applications in regenerative medicine, tissue engineering, and cell therapy are expected. Related studies have shown that this technology demonstrates good results in improving cell survival and promoting angiogenesis, providing a new solution for clinical cell transplantation.

#### 2.3.3 Microvascular fragments (MVFs) constructing adipose organoids

Microvascular fragments (MVFs), as an important biological material, show great application potential in tissue engineering. MVFs are functional vascular units extracted from adipose tissue, containing microvascular networks that can rapidly reorganize and form new microvascular systems *in vivo* (Laschke and Menger, 2022).

In the research on MVFs, scientists have discovered their ability to enhance angiogenesis and promote adipose tissue regeneration. Specifically, by providing an appropriate three-dimensional culture environment for MVFs, they can self-assemble into microvascular networks, which is crucial for constructing adipose organoids. For example, utilizing a collagen-based matrix for three-dimensional culture can significantly enhance the angiogenesis ability of MVFs, promoting the differentiation and survival of adipocytes, thereby forming more complex adipose structures (Salamone et al., 2021).

In improving skin fibrosis, MVFs have also shown positive effects. Studies indicate that MVFs can improve the regenerative environment of skin tissue by promoting local blood supply and nutrient delivery, thereby reducing the incidence of fibrosis (Quan et al., 2023). This mechanism primarily involves enhancing the formation of new blood vessels, improving the local microenvironment, and promoting collagen reconstruction and regeneration, making clinical applications in treating skin injuries and chronic wounds.

Overall, the study of forming adipose organoids through three-dimensional culture of MVFs has greatly advanced the progress of regenerative medicine. They not only provide a good angiogenesis foundation for adipose tissue regeneration but also demonstrate good application prospects in improving skin fibrosis. In the future, with further in-depth research on the characteristics of MVFs, their clinical applications will become more widespread, providing new solutions for addressing various tissue defects and regeneration issues.

### 2.4 Biomaterials and scaffold optimization

#### 2.4.1 Porous polycaprolactone (PCL) composite scaffolds

Porous polycaprolactone (PCL) composite scaffolds exhibit outstanding application potential in the field of tissue engineering, particularly in promoting angiogenesis and inhibiting fibrosis (Liu et al., 2022). PCL, a polymer with good biocompatibility, is widely used in the preparation of scaffolds due to its significant mechanical strength and excellent biodegradability, making it an ideal choice for bone tissue engineering.

Studies have shown that PCL composite scaffolds possess an excellent porous structure that can effectively support cell growth and migration. The porosity of such scaffolds typically exceeds 90%, providing an ideal growth environment for cells and facilitating the transfer of nutrients and oxygen, which is crucial for cell survival and function (Shi et al., 2022). In vitro experiments, PCLMF composite scaffolds significantly enhanced the adhesion rate and proliferation ability of bone marrow mesenchymal stem cells (BMSCs), with a noticeable improvement in osteogenic capability compared to other materials. 3D-printed PCL scaffolds loaded with bFGF and BMSCs promoted tendon-bone healing after rotator cuff tears in rats, demonstrating good biocompatibility and regenerative ability in preclinical studies (Ni et al., 2025). By implanting this scaffold in a mouse model of bone defects, results indicated that the scaffold could effectively promote vascular regeneration, increase the rate of new bone formation, and significantly reduce the occurrence of fibrosis during the tissue healing process (Park et al., 2024; Song et al., 2024). This effect is closely related to the scaffold’s superior porous structure, reasonable mechanical strength, and good cell compatibility.

In summary, the superior performance of porous PCL composite scaffolds in promoting angiogenesis and reducing fibrosis shows promising application prospects in tissue engineering, particularly in the field of bone tissue regeneration. This scaffold not only provides a “home” for cell growth but also enhances the overall effect of tissue regeneration through its excellent biomechanical properties and biocompatibility, providing strong support for future clinical applications.

#### 2.4.2 Decellularized adipose tissue hydrogel

Decellularized adipose tissue hydrogel (DAT Hydrogel) is a biomaterial that shows great potential in soft tissue engineering. With the development of tissue engineering, the application of decellularized adipose tissue as scaffold material has been extensively studied due to its rich source and excellent potential in tissue regeneration (Xiong et al., 2024). This hydrogel not only provides a three-dimensional microenvironment that supports cell growth but also promotes angiogenesis and the regeneration of adipose tissue through its natural extracellular matrix (ECM) components.

Research has shown that decellularized adipose tissue hydrogel can effectively support the release of adipose tissue-derived extracellular vesicles (ATEVs), which play an important role in promoting angiogenesis and adipogenesis. In one study, researchers utilized a novel mechanical separation technique to extract vesicles from adipose liquid and prepared an exosome-rich decellularized adipose tissue hydrogel. This hydrogel demonstrated strong angiogenic and adipogenic capabilities when co-cultured with vascular endothelial cells and adipose progenitor cells (Nie et al., 2021). In mouse models, the injection of this exosome-enriched hydrogel resulted in significant increases in the volume and angiogenesis of newly formed tissue, indicating its broad application prospects in soft tissue regeneration.

Moreover, the preparation methods for decellularized adipose tissue hydrogel are continually being optimized. For example, using enzyme-free methods for decellularization not only effectively removes adipocytes but also preserves the microstructure, thereby better supporting cell adhesion and growth. In vitro cultures, decellularized adipose matrix (DAM) has shown good cell compatibility, capable of supporting the proliferation and differentiation of human adipose-derived stem cells (hADSCs) and promoting the secretion of VEGF (Qi et al., 2024).

Regarding clinical applications, studies have begun to explore the use of decellularized adipose tissue hydrogel in soft tissue repair and regeneration. For instance, in breast reconstruction surgeries, combining biodegradable scaffolds with decellularized adipose tissue can effectively improve the volume and angiogenesis of regenerated fat, providing better treatment options for patients (Zhang et al., 2022). These findings indicate that decellularized adipose tissue hydrogel has significant application potential in soft tissue engineering and is gradually becoming an ideal material choice. Through further research and clinical trials, decellularized adipose tissue hydrogel is expected to play an increasingly important role in future tissue regeneration and repair.

#### 2.4.3 Collagen sponge scaffolds in islet transplantation

In the field of islet transplantation, collagen sponge scaffolds as a biomaterial show good application prospects. Their main function is to provide a microenvironment that supports and promotes cell growth, significantly enhancing the success rate of islet transplantation when combined with growth factors such as bFGF. Research has shown that bFGF plays a crucial role in enhancing angiogenesis and promoting cell proliferation, especially in transplant environments lacking good blood supply, effectively promoting the survival and functional maintenance of transplanted islets (Nakafusa et al., 2022).

Firstly, the biocompatibility of collagen sponge makes it an ideal scaffold material. Compared to traditional materials, collagen sponge better mimics the extracellular matrix (ECM), providing necessary support for islet cells and promoting their growth and functional recovery (Wu et al., 2024).

**FIGURE 2:**
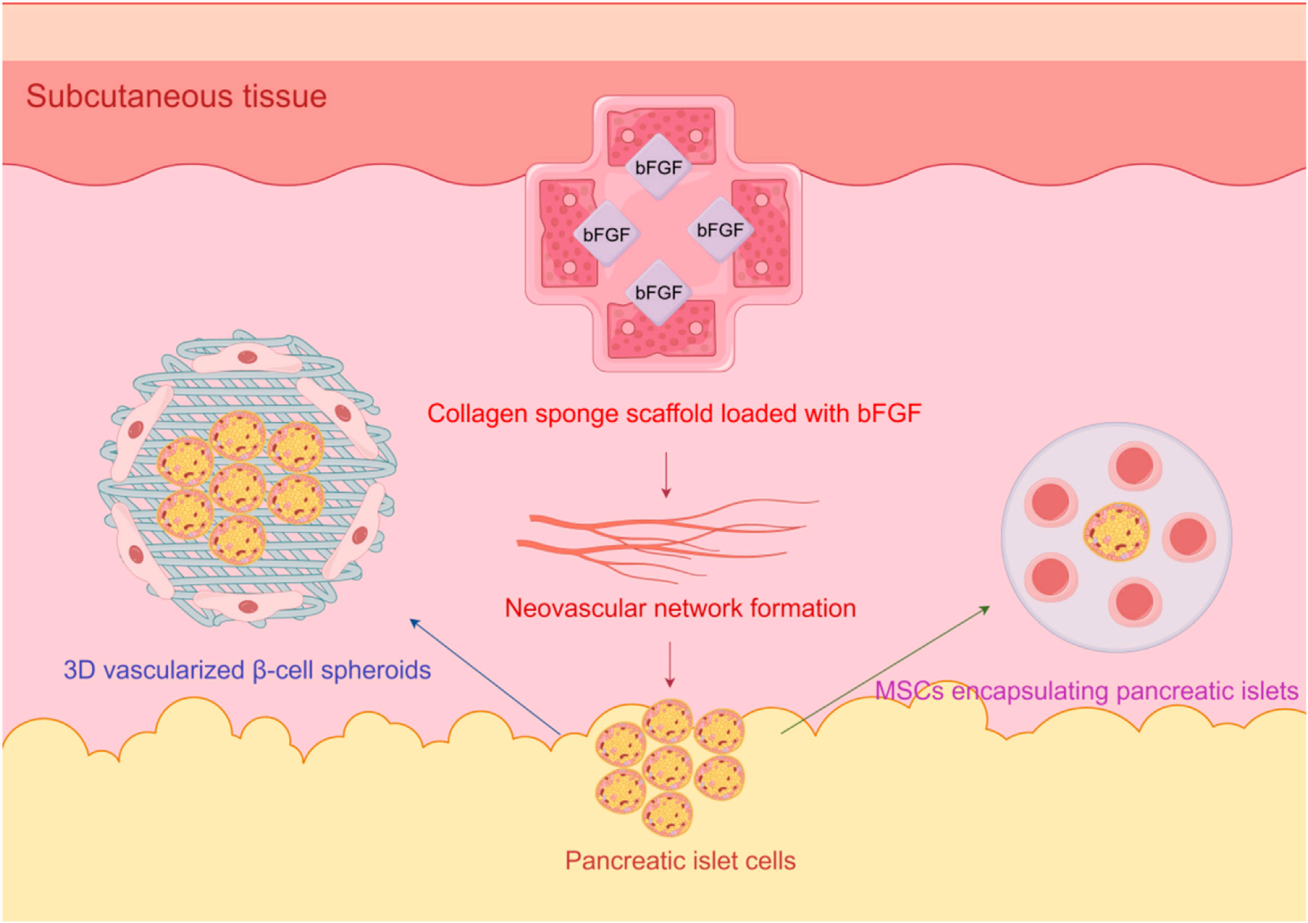
Schematic of angiogenesis optimization in subcutaneous islet transplantation for diabetes, integrating 3D vascularized β-cell spheroids, bFGF-loaded collagen sponges, and MSC encapsulation.

Secondly, the use of bFGF further enhances the effectiveness of collagen sponge. In the environment of collagen sponge, the release of bFGF can be sustained and stable, effectively supporting the revascularization of islets after transplantation. Existing studies have shown that the application of bFGF can significantly improve the function of transplanted islets, lower blood glucose levels, and promote blood vessel formation, thereby increasing the success rate of transplantation (Gu et al., 2001). Figure 2 illustrates the synergistic strategies for subcutaneous islet transplantation:3D β-cell spheroids activate angiogenic pathways via paracrine factors, bFGF-loaded collagen sponges induce neovascular networks, and MSCs improve the microenvironment to suppress immune rejection.

Additionally, the use of collagen sponge scaffolds can effectively control the release of bFGF through a sustained release system, maintaining its biological activity for a long time after transplantation, thereby better supporting the angiogenesis and functional recovery of the graft. This approach not only improves the survival rate of islets but also reduces the occurrence of postoperative complications (Emoto et al., 2025).

In summary, the application of collagen sponge scaffolds combined with bFGF in islet transplantation shows promising prospects, providing new ideas and methods to improve the success rate and clinical efficacy of islet transplantation.

### 2.5 The relationship between immunoregulation and angiogenesis

#### 2.5.1 The impact of macrophage polarization on angiogenesis

During inflammation and tissue repair, the polarization state of macrophages plays a critical role in angiogenesis (Xie et al., 2022). Macrophages can polarize into M1 or M2 types depending on their microenvironment. M1 macrophages are typically associated with pro-inflammatory responses and can produce various pro-inflammatory cytokines, while M2 macrophages are considered to have anti-inflammatory and tissue repair-promoting functions (Zhang et al., 2018). Studies have shown that M2 macrophages significantly promote angiogenesis and tissue regeneration by secreting various growth factors and cytokines (Yang M. et al., 2025).

One study found that M2 macrophages can promote angiogenesis not only by secreting VEGF but also by modulating the migration and proliferation of endothelial cells, further enhancing the capacity for vascular formation. Moreover, M2 macrophages support tissue repair and regeneration processes by releasing anti-inflammatory factors and promoting matrix remodeling, which is particularly important in chronic diseases such as diabetic wound healing (Yang M. et al., 2025).

MSCs also play an important role in promoting M2 macrophage polarization. Research has shown that MSCs can effectively induce macrophage polarization toward M2 type by secreting the cytokine IL-10, thereby enhancing their anti-inflammatory and repair-promoting functions (Aktas et al., 2017; Gong et al., 2025). Therefore, the interaction between MSCs and macrophages plays a key regulatory role in tissue repair and angiogenesis.

In the fields of stem cell therapy and regenerative medicine, the strategy of using MSCs to induce M2 macrophage polarization has shown positive preclinical effects. This provides new ideas for developing novel therapeutic approaches, especially in chronic wound healing and tissue regeneration, where the interaction between MSCs and macrophages will be a focus of future research (Bejugam et al., 2025). By understanding the mechanisms of macrophage polarization, we can better utilize these cells in clinical applications, promoting the development of regenerative medicine and tissue engineering.

### 2.6 Subcutaneous angiogenesis in islet transplantation

#### 2.6.1 The angiogenic needs and challenges of islet cells

The angiogenic needs and challenges of islet cells are particularly important in the field of islet transplantation (Rojas-Canales et al., 2017). The survival and function of islet cells depend on effective blood supply, while the vascular network in subcutaneous tissue is sparse, posing significant challenges for islet cell transplantation (Jeon et al., 2024; Emoto et al., 2025). In the subcutaneous tissue, insufficient angiogenesis leads islet cells to face risks of hypoxia and malnutrition after transplantation, directly affecting their functional maintenance and the therapeutic outcomes for diabetic patients.

Research has shown that the success of islet cell transplantation is closely related to the angiogenesis level at the transplantation site (Jansson and Carlsson, 2002). Compared to traditional islet transplantation methods, the advantage of subcutaneous transplantation lies in its minimally invasive nature and ease of monitoring, but its limitation is insufficient angiogenesis, resulting in high islet cell mortality and rapid functional decline (Shi et al., 2016; Yu et al., 2020). In this context, developing strategies to promote angiogenesis becomes particularly important.

By optimizing the microenvironment of islet cells, researchers have explored a series of methods to promote angiogenesis. For instance, scaffolds constructed from biocompatible materials can effectively promote angiogenesis at the transplantation site, thereby improving the survival rate and function of islet cells (Emoto et al., 2025). Additionally, studies have found that combining the transplantation of MSCs with islet cells can significantly enhance post-transplant angiogenesis and improve islet cell function, providing new ideas for clinical treatment (Pinheiro-Machado et al., 2025).

From a cellular and molecular perspective, key factors promoting angiogenesis in islet cells include VEGF and bFGF (Wu et al., 2024). These factors can stimulate the proliferation and migration of endothelial cells, thereby promoting the formation of new blood vessels (Luo et al., 2011; Kado et al., 2024). Other studies have shown that using a strategy combining extracellular matrix scaffolds and growth factors can effectively improve the microenvironment of islet cells, enhancing their survival capability and function (Wang X. et al., 2023).

In summary, although the sparse vascular network in subcutaneous tissue poses challenges to the survival and function of islet cells, scientific interventions and emerging biomaterials technology hold promise for overcoming these limitations, enhancing the success rate and clinical efficacy of islet cell transplantation. Future research should further explore the effects of different combinatorial strategies on islet cell angiogenesis to achieve more effective treatment options for diabetes.

#### 2.6.2 Strategies for promoting angiogenesis with cells and materials

In recent years, strategies involving cells and materials to promote angiogenesis have been widely applied in tissue engineering and regenerative medicine, especially in the treatment of organ transplantation and diseases such as diabetes. To improve transplantation outcomes, researchers have developed various combinations of biomaterials and cells, among which 3D vascularized β-cell spheroids, collagen sponge carriers, and delivery strategies for bFGF have shown significant effects.

3D vascularized β-cell spheroids are a novel cell structure that can provide a physiologically relevant microenvironment, aiding in the survival and function of β-cells. Research has shown that this three-dimensional structure not only improves cell-to-cell interactions but also promotes angiogenesis, thereby enhancing cell survival rates and function (Fukuda et al., 2018; Takaichi et al., 2022). Promoting the rapid fabrication and functionality of pancreatic β-cell spheroids allows transplanted β-cells to integrate more efficiently with host tissue, reducing cell apoptosis and improving therapeutic outcomes.

As an ideal biomaterial, collagen sponge scaffolds are widely used in tissue engineering due to their superior biocompatibility and biodegradability. Collagen sponges not only support cell attachment, proliferation, and differentiation but also provide necessary physical support. In studies, the combined use of collagen sponge scaffolds with β cells not only promotes cell growth but also improves transplant outcomes by enhancing local angiogenesis (Wu et al., 2024). The application of this scaffold offers a more suitable microenvironment for cells, helping to enhance the function and survival rate of transplanted tissues.

In addition, bFGF, as an important growth factor, plays a significant role in promoting angiogenesis. It enhances the formation of new blood vessels by activating multiple signaling pathways that stimulate the proliferation and migration of endothelial cells. Studies have shown that bFGF can effectively stimulate angiogenesis, improve blood supply to the transplant area, and thus increase the survival rate and function of β cells (Jansson and Carlsson, 2002).

In summary, the application of three-dimensional vascularized β cell spheroids, collagen sponge carriers, and bFGF provides new perspectives for promoting angiogenesis and improving cell transplantation outcomes. These strategies not only increase cell survival rates but also promote angiogenesis, providing important support for tissue regeneration and functional recovery. These research findings lay a solid theoretical foundation and practical guidance for future clinical applications.

#### 2.6.3 Clinical translation prospects and existing issues

With the rapid development of regenerative medicine and tissue engineering, low-immunogenic materials and technologies that promote angiogenesis are gradually showing promise in clinical applications, but they also face many challenges. The development of low-immunogenic materials provides an important foundation for tissue transplantation, as they can effectively reduce the rejection response after organ or tissue transplantation, thereby improving the success rate of transplants. For example, the use of highly biocompatible materials, such as polylactic acid (PLA) and polyvinyl alcohol (PVA), has been shown to reduce immune responses during the transplantation process and promote tissue regeneration (Pinto et al., 2010; Yang et al., 2020).

On the other hand, technologies that promote angiogenesis have shown great potential in various clinical applications, especially in the field of tissue regeneration where vascular supply is urgently needed. By using growth factors, cell therapy, or synthetic materials, researchers are able to effectively promote the formation of new blood vessels, thereby improving the blood supply to tissues. For example, the application of bFGF has been proven to significantly enhance angiogenesis, thus increasing the survival rate and function of transplanted tissues (Emoto et al., 2025). In addition, engineered extracellular vesicles (EVs) as an emerging biomaterial can promote angiogenesis by transporting bioactive factors, showing promising prospects for clinical translation (Tu et al., 2023).

However, despite the broad prospects, clinical translation still faces multiple challenges. First, the long-term effects and biocompatibility of low-immunogenic materials still require substantial clinical data support; current research is largely focused on animal experiments, lacking large-scale human clinical trials. Second, strategies for promoting angiogenesis may exhibit significant differences among different patients and types of tissues, making the development of personalized treatment plans particularly necessary.

Additionally, there are issues of cost and challenges in production standardization in clinical translation. The research and development of new materials and technologies often require high costs, which may be difficult for existing healthcare systems to bear. Moreover, ensuring quality control of these materials during the manufacturing process and the safety of their clinical applications is also a critical issue that cannot be overlooked (Metselaar and Lammers, 2020).

In summary, although low-immunogenic materials and angiogenesis-promoting technologies show good prospects for clinical application, achieving widespread use still requires overcoming issues related to immune response, individual differences, cost, and standardization. Future research should focus on gaining a deeper understanding of the mechanisms of these technologies, optimizing clinical trial designs to facilitate their successful clinical translation.

### 2.7 Applications of subcutaneous angiogenesis in soft tissue repair and autoimmune diseases

#### 2.7.1 Adipose tissue engineering and angiogenesis

Adipose tissue engineering plays a crucial role in soft tissue reconstruction, and its success mainly depends on effective angiogenesis. In recent years, researchers have gradually recognized the important role of adipose tissue-derived extracellular vesicles (ATEVs) in promoting angiogenesis and improving adipose tissue survival (Chen et al., 2022). ATEVs are small extracellular vesicles released by cells derived from adipose tissue, rich in various bioactive molecules that can effectively regulate intercellular signaling, promote the proliferation and migration of endothelial cells, thereby enhancing the capacity for angiogenesis (Nie et al., 2021).

One study proposed a novel mechanical separation technique, and ATEVs extracted through this technique were shown to effectively promote the proliferation of endothelial cells and lumen formation *in vitro*. In vivo experiments, ATEV-enriched hydrogels significantly increased the volume of adipose tissue, and both angiogenesis and adipogenesis were improved. In the experimental group injected with ATEV-enriched hydrogel, higher angiogenesis and adipogenesis were observed at the 4th and 8th weeks post-surgery (Nie et al., 2021). Furthermore, employing improved composite hydrogel strategies, such as combining decellularized adipose tissue (DAT) with small intestinal submucosa (SIS) or adventitia (Adv), can effectively promote new blood vessel formation and adipose tissue regeneration, further indicating the application potential of composite hydrogels in adipose tissue engineering (Cui et al., 2023).

In the process of adipose transplantation, delayed and unstable angiogenesis is a key factor affecting adipose survival. By optimizing extracellular vesicle application and combining them with bioactive hydrogels, the angiogenesis of transplanted adipose can be enhanced and their survival rate improved, thus providing new solutions for clinical applications (Zhu et al., 2022). For example, hydrogels combined with extracellular vesicles can provide necessary growth factors in the early postoperative period, promoting blood vessel formation and subsequently reducing adipose tissue necrosis rates, improving overall regenerative outcomes (Xie et al., 2020).

In summary, adipose tissue-derived extracellular vesicles and their composite hydrogels exhibit good angiogenesis-promoting capabilities and improvements in adipose survival in adipose tissue engineering. Future research can further explore combinations of different types of extracellular vesicles and hydrogels to optimize strategies for adipose tissue engineering, thereby addressing clinical challenges in soft tissue reconstruction more effectively.

#### 2.7.2 Regulation of angiogenesis in local fibrotic diseases

One characteristic of local fibrotic diseases is the loss of subcutaneous fat and abnormal fibrosis, commonly seen in conditions like localized scleroderma. Recent studies have shown that three-dimensional (3D) cultured adipose organoids can effectively restore subcutaneous fat and reduce the extent of local fibrosis. Specifically, adipose-derived stem cells (ADSCs) have demonstrated positive therapeutic effects in local fibrotic models; these cells not only promote fat regeneration but also alleviate fibrosis by modulating inflammatory responses and enhancing angiogenesis (Wang et al., 2023b).

In a study involving a mouse model of localized scleroderma, researchers injected different doses of ADSCs into the subcutaneous tissue of mice. Results indicated that high doses of ADSCs significantly reduced skin fibrosis, lowered the production of type III collagen and transforming growth factor β1 (TGF-β1), and increased the expression of cytokines related to angiogenesis. This suggests that the application of ADSCs can not only promote fat retention but also improve the local microenvironment by promoting angiogenesis, thereby alleviating fibrosis (Wang et al., 2023b).

Additionally, another study found that dedifferentiated adipocytes (DAs) exhibited stronger activity in inducing angiogenesis and effectively suppressed the expression of fibrosis-related genes. These findings provide new insights into the treatment of local fibrotic diseases, indicating that the combination of 3D culture techniques with the application of ADSCs or DAs may achieve fat tissue regeneration and functional repair, thus improving pathological states (Wang et al., 2022).

In designing treatment plans, consideration should also be given to the modulation of the local microenvironment, such as through the combined application of platelet-rich plasma (PRP) and fat transplantation to further enhance angiogenesis and fat survival. This strategy has shown significant clinical effects in studies, not only alleviating skin fibrosis but also increasing the survival rate of fat transplants, further validating the potential of 3D cultured adipose organoids in treating local fibrosis (Wang et al., 2023a).

In summary, 3D cultured adipose organoids provide new mechanisms and strategies for restoring subcutaneous fat and reducing local fibrosis. Future research can further explore the effects of different cell sources and their combined applications on local fibrotic diseases, aiming to provide more effective therapeutic options for clinical practice. The regulation of angiogenesis is regarded as an important strategy for improving the local immune environment. Autologous fat transplantation, as a regenerative medicine technology, has been widely applied in the treatment of various diseases clinically, especially in those related to angiogenesis.

## 3 Conclusion

Subcutaneous angiogenesis in transplantation is a key factor influencing transplantation success, and the development history and research progress reveal that complex molecular signals and cellular interactions play an important role in this process. In recent years, significant achievements have been made in the study of subcutaneous angiogenesis through various strategies, including growth factor delivery, stem cell co-transplantation, biomaterial optimization, and immune regulation. These strategies not only enhance the survival and function of transplanted cells but also provide broad application prospects in fields such as islet transplantation, cancer treatment, soft tissue repair, and autoimmune diseases (Table 1).

**TABLE 1:** Recent advances in biomaterials for promoting angiogenesis in subcutaneous transplantation.

Material category	Specific examples	Key features and mechanisms	Recent advancements and applications
Natural Polymer Scaffolds	Gelatin Hydrogel Nonwoven Fabric (GHNF)	Provides three-dimensional support, augments the extracellular matrix (such as laminin, collagen III/IV), and enhances the microenvironment conducive to the survival of transplanted islets; however, its ability to promote angiogenesis is constrained and does not primarily entail direct stimulation of substantial neovascularization	Combined with adipose-derived stem cells (ADSCs) to significantly enhance the engraftment and function of subcutaneous islets (Kanai et al., 2023; Saito et al., 2023a; Saito et al., 2024)
	Collagen Sponge	Exhibiting outstanding biocompatibility, it emulates the intrinsic extracellular matrix (ECM) and functions as a sustained-release vehicle for growth factors such as bFGF.	Loaded with bFGF, this preparation successfully facilitates the induction of functional neovascular networks in subcutaneous islet transplantation, thereby reversing hyperglycemia (Nakafusa et al., 2022; Wu et al., 2024; Emoto et al., 2025)
	Decellularized Adipose Tissue Hydrogel (DAT Hydrogel)	Preserves natural adipose ECM components and bioactive factors, exhibiting excellent pro-angiogenic and adipogenic capabilities; can be prepared via enzyme-free decellularization to retain microstructure for better cell adhesion/growth	Integration with adipose tissue-derived extracellular vesicles (ATEVs) or combination with small intestinal submucosa (SIS) to construct highly vascularized adipose tissue (Nie et al., 2021; Cui et al., 2023; Qi et al., 2024)
Synthetic Polymer Scaffolds	Porous Polycaprolactone (PCL)	Excellent biocompatibility, adjustable biodegradability, and robust mechanical strength; elevated porosity promotes cellular migration and nutrient transport	3D-printed composite scaffolds infused with bFGF and bone marrow-derived mesenchymal stem cells (BMSCs) facilitate the healing of tendon-bone junctions through immunomodulatory mechanisms and the promotion of angiogenesis (Park et al., 2024; Song et al., 2024; Ni et al., 2025)
	Polylactic Acid (PLA)/Polyglycolic Acid (PGA)	Biodegradable and approved by the FDA for certain medical devices, it has the potential to function as a controlled-release mechanism for growth factors or pharmaceuticals	Utilized as carriers with low immunogenicity for the localized administration of immunosuppressive agents or growth factors, aimed at minimizing rejection and enhancing vascularization (Pinto et al., 2010; Yang et al., 2020)
Composite	Nanogels	High drug-loading capacity, capable of intelligent, stimuli-responsive (e.g., pH, temperature, tumor microenvironment) drug release; temperature-responsive types (e.g., PNIPAM-based) regulate swelling for controlled release	Emerging as innovative delivery mechanisms for angiogenic agents (e.g., VEGF); possess significant promise for spatiotemporal modulation of vascularization (Niezabitowska et al., 2023; Gülyüz et al., 2024; Vashist et al., 2024)
	Engineered Extracellular Vesicles (EVs)	Natural intercellular communication vehicles, which can be equipped with specific proteins, RNAs, and other molecular entities, exhibit low immunogenicity	Designed to enhance pro-angiogenic factors and employed as a “cell-free” therapeutic approach in conjunction with scaffolds for the meticulous modulation of angiogenesis (Tu et al., 2023)
Bioactive Factor Delivery Systems	bFGF-Chitosan Gel	The cationic characteristics of chitosan facilitate the stable attachment and prolonged release of bFGF.	Activated endogenous neurogenesis and angiogenesis in a rat model of ischemic stroke (Duan et al., 2024)
	Platelet-Rich Plasma (PRP)	Of autologous origin and abundant in various growth factors (VEGF, PDGF, TGF-β), it is safe and readily obtainable. Its efficacy is influenced by preparation techniques (e.g., activation protocols) and platelet concentration	Combined with fat grafting significantly improves fat survival and accelerates diabetic wound healing by upregulating pathways like Notch1 (Kakudo et al., 2014; Zhou et al., 2017)

Nevertheless, the regulatory mechanisms of angiogenesis still hold many unanswered questions, and researchers face multiple challenges in exploring these complex mechanisms, including immune rejection responses and clinical translation. The viewpoints and findings among different studies often vary, which requires us to maintain a rigorous and objective attitude in our analyses. Regarding the effectiveness of certain strategies, although multiple experimental results support their feasibility, attention must also be paid to their adaptability and limitations under different physiological and pathological conditions. For instance, stem cell co-transplantation may not promote angiogenesis as expected under specific conditions, necessitating a meticulous consideration of the interactions of various factors in future research.

Future research directions should focus on deepening the understanding of molecular mechanisms while optimizing synergistic strategies involving multiple components to address the current challenges. Through detailed mechanistic studies, we will be better able to identify and utilize key regulatory factors to enhance the efficiency of angiogenesis. Additionally, developing novel immune regulation strategies is particularly important to address immune rejection responses, which will lay the groundwork for clinical translation. A good foundation.

In the process of promoting the clinical application of subcutaneous angiogenesis technology, we also need to pay attention to the overall therapeutic effects in the fields of tissue engineering and regenerative medicine. Interdisciplinary collaboration, technological innovation, and clinical feedback will be the driving forces for continuous progress in this field. In summary, although current research has provided various effective strategies for subcutaneous angiogenesis, continuous efforts are still needed in understanding mechanisms, optimizing strategies, and clinical translation to achieve better clinical outcomes and patient welfare.

## Abbreviations

VEGF, Vascular Endothelial Growth Factor; ADSCs, Adipose-Derived Stem Cells; GHNF, Gelatin Hydrogel Nonwoven Fabric; bFGF, Basic Fibroblast Growth Factor; HIF, Hypoxia-Inducible Factor; MSCs, Mesenchymal Stem Cells; VEGFA, Vascular Endothelial Growth Factor A; VEGFR2, Vascular Endothelial Growth Factor Receptor 2; PI3K/Akt, Phosphatidylinositol 3-Kinase/Protein Kinase B; MAPK/ERK, Mitogen-Activated Protein Kinase/Extracellular Signal-Regulated Kinase; ARL13B, ADP-Ribosylation Factor-Like Protein 13B; HUVECs, Human Umbilical Vein Endothelial Cells; PRP, Platelet-Rich Plasma; PDGF, Platelet-Derived Growth Factor; TGF-β, Transforming Growth Factor β; MMPs, Matrix Metalloproteinases; EGF, Epidermal Growth Factor; EVs, Extracellular Vesicles; MVFs, Microvascular Fragments; PCL, Polycaprolactone; BMSCs, Bone Marrow Mesenchymal Stem Cells; DAT Hydrogel, Decellularized Adipose Tissue Hydrogel; ATEVs, Adipose Tissue-Derived Extracellular Vesicles; DAM, Decellularized Adipose Matrix; ECM, Extracellular Matrix; IL-10, Interleukin 10; PLA, Polylactic Acid; PVA, Polyvinyl Alcohol; SIS, Small Intestinal Submucosa; Adv, Adventitia; DAs, Dedifferentiated Adipocytes.
